# Type-2 diabetes-induced changes in vascular extracellular matrix gene expression: Relation to vessel size

**DOI:** 10.1186/1475-2840-5-3

**Published:** 2006-02-17

**Authors:** WeiWei Song, Adviye Ergul

**Affiliations:** 1 Program in Clinical and Experimental Therapeutics, the University of Georgia College of Pharmacy, Augusta, Georgia 30912, USA; 2 Vascular Biology Center, Medical College of Georgia, Augusta, Georgia 30912, USA

## Abstract

**Background:**

Hyperglycemia-induced changes in vascular wall structure contribute to the pathogenesis of diabetic microvascular and macrovascular complications. Matrix metalloproteinases (MMP), a family of proteolytic enzymes that degrade extracellular matrix (ECM) proteins, are essential for vascular remodeling. We have shown that endothelin-1 (ET-1) mediates increased MMP activity and associated vascular remodeling in Type 2 diabetes. However, the effect of Type 2 diabetes and/or ET-1 on the regulation of ECM and MMP gene expression in different vascular beds remains unknown.

**Methods:**

Aorta and mesenteric artery samples were isolated from control, Type 2 diabetic Goto-Kakizaki (GK) rats and GK rats treated with ET_A _antagonist ABT-627. Gene expression profile of MMP-2, MMP-9, MT1-MMP, fibronectin, procollagen type 1, c-fos and c-jun, were determined by quantitative real-time (qRT) PCR. In addition, aortic gene expression profile was evaluated by an ECM & Adhesion Molecules pathway specific microarray approach.

**Results:**

Analysis of the qRT-PCR data demonstrated a significant increase in mRNA levels of MMPs and ECM proteins as compared to control animals after 6 weeks of mild diabetes. Futhermore, these changes were comparable in aorta and mesentery samples. In contrast, treatment with ET_A _antagonist prevented diabetes-induced changes in expression of MMPs and procollagen type 1 in mesenteric arteries but not in aorta. Microaarray analysis provided evidence that 27 extracellular matrix genes were differentially regulated in diabetes. Further qRT-PCR with selected 7 genes confirmed the microarray data.

**Conclusion:**

These results suggest that the expression of both matrix scaffold protein and matrix degrading MMP genes are altered in macro and microvascular beds in Type 2 diabetes. ET_A _antagonism restores the changes in gene expression in the mesenteric bed but not in aorta suggesting that ET-1 differentially regulates microvascular gene expression in Type 2 diabetes.

## Introduction

Changes in vascular wall structure occur in diabetes and contribute to both micro- and macrovascular complications. Previous studies in streptozosin (STZ)-induced model of Type 1 diabetes documented increased intimal proliferation and medial thickness as well as extracellular matrix (ECM) deposition in microvessels such as mesenteric arteries as early as 3 weeks of experimental diabetes [1-4]. Vascular remodeling and hypertrophy associated with augmented expression of dedifferentiation markers of vascular smooth muscle cells also occur in larger vessels like aorta [5]. While these studies provided evidence for diabetes-induced alterations in ECM synthesis and vascular structure of an experimental model of Type 1 diabetes that is characterized by highly elevated blood glucose levels, to what extent mild-to-modest hyperglycemia as seen in Type 2 diabetes influences the gene expression of ECM proteins associated with vascular remodeling and whether there are differences in micro vs macrovascular bed are not fully understood.

Vascular ECM proteins such as collagen type 1 and 3, fibronectin and thrombospondins not only function as scaffolding proteins but also involved in matrix signaling by interacting with integrin family of proteins and triggering growth-promoting signals. ECM displays a very dynamic equilibrium where there is constant synthesis, degradation and reorganization. Turnover of matrix proteins are regulated by matrix metalloproteinases (MMPs) [6]. While decreased MMP activity is generally believed to contribute to ECM accumulation in diabetic kidney and in vascular tissue from patients with diabetes, we and others have recently reported that there is an early activation of MMPs in hypertension and diabetes [7-9]. However, transcriptional regulation of ECM proteins and MMPs in different vascular beds and specifically in Type 2 diabetes remains to be determined.

Vasoactive factors including endothelin-1 (ET-1) and angiotensin II are involved in diabetic vascular remodeling as evidenced by studies that demonstrated attenuation of these responses by blockade of these systems in both experimental and clinical diabetes. For example, Gilbert and colleagues reported that ET_A _receptor antagonism prevents mesenteric vascular hypertrophy in Type 1 diabetes [4]. Another study provided evidence that blockade of ET-1 action inhibits ECM deposition in the aorta as well [5]. We recently reported that ET-1 levels are elevated and an ET_A _antagonist prevents ECM deposition and MMP activation in middle cerebral arteries but not in the kidney of Goto-Kakizaki (GK) rats, a non-obese Type 2 diabetes model [9,10]. Thus, this study was designed to test the hypothesis that there is a differential regulation of MMP activation in micro vs macrovessels in Type 2 diabetes and ET-1 contributes to this process.

## Methods

### Animal and tissue preparation

All experiments were performed on male Wistar (Harlan, Indianapolis, IN) and Goto-Kakizaki (in-house bred, derived from the Tampa colony) rats [11]. The animals were housed at the Medical College of Georgia animal care facility that is approved by the American Association for Accreditation of Laboratory Animal Care and study was approved by the Institutional Animal Care and Use Committee. Animals were fed standard rat chow and tap water ad-libitum. During housing, drinking water measurements, weight, and blood glucose measurements were performed twice weekly. At 12 weeks of age, when all GK animals became overtly diabetic, telemetry transmitters for blood pressure measurements were implanted as previously reported [12]. After a 2 week-recovery period, control and diabetic animals were administered the ET_A _selective antagonist, ABT-627 (5 mg/kg/day) in drinking water, or vehicle only [8]. Treatment was maintained until the time of sacrifice at 18 weeks of age. Animals were anesthetized with sodium pentobarbital and exsanguinated via the abdominal aorta. Upon sacrifice, the mesenteric bed was harvested, third order mesenteric arteries and thoracic aorta were isolated and immediately put into RNA*later*™ (Ambion, Austin, TX, USA) for storage at -80°C.

### RNA isolation and cDNA synthesis

Total RNA extraction was carried out using the RNeasy^® ^Mini kit (Qiagen Inc., Valencia, CA) according to manufacturer's instructions. RNA quality from each sample was assured by the A260/280 absorbance ratio and by electrophoresis of 1.2% agarose formaldehyde gel. 1.0–2.0 μg of total RNA was reverse transcribed into single strand cDNA using MuLV reverse transcriptase (Applied Biosystems, Foster City, CA, USA). RT reaction was carried out for 60 min at 42°C and 5 min at 95°C in a thermocycler.

### Primer design and qRT-PCR

All oligonucleotide primer sets were designed based upon published mRNA sequence. Expected amplicon lengths were from 100 bp – 200 bp. Oligonucleotide primers used in this study are listed in Table 1. The qRT-PCR was performed in a SmartCycler II (Cepheid, Sunnyvale, CA) by using SYBR^® ^Green PCR Master Mix (Applied Biosystems, Foster City, CA, USA). 2–2.5 μl of cDNA template was used for qRT-PCR in a final volume of 25 μl. cDNA was amplified according to the following condition: 95°C for 15 s and 60°C for 60 s from 40 to 45 amplification cycles. Fluorescence changes were monitored with SYBR Green after every cycle. Melting curve analysis was performed (0.5°C/s increase from 55–95°C with continuous fluorescence readings) at the end of cycles to ensure that single PCR products were obtained. Amplicon size and reaction specificity were confirmed by 2.5% agarose gel electrophoresis. All reactions were repeated in 3 separate PCR runs using RNA isolated from 3 sets of animals. Results were evaluated with the SmartCycler II software. Glyceraldehyde-3-phosphate dehydrogenase (GAPDH) primers were used to normalize samples. To monitor crossover contaminations of PCR, RNase-free water (Qiagen Inc., Valencia, CA) was included in the RNA extraction and used as a negative control. To ensure quality of data, a negative control was always applied in each run.

**Table 1:** List of primers used in qRT-PCR

Primer ID	Accession No	Sequence	Expect Size (bp)
MMP-9 (F)	NM_031055	GTC TTC CCC TTC GTC TTC CT	117
MMP-9 (R)		ACC CCA CTT CTT GTC AGC GT	
MMP-14 (F)	NM_031056	TGT CCC AGA TAA GCC CAG AA	128
MMP-14 (R)		TAT TCC TCA CCC GCC AGA AC	
GAPDH (F)	XM_234433	AGC CCA GAA CAC CAT TCC TAC	191
GAPDH (R)		ATG CCT GCT TCA CCA CAT TC	
FIBRONECTIN (F)	X15906	GCA CAG GGG AAG AAA AGG AG	189
FIBRONECTIN (R)		TTG AGT GGA TGG GAG GAG AG	
MMP-2 (F)	NM_031054	GTG CCA AGG TGG AAA TCA GAG	110
MMP-2 (R)		AAG GTT GAA GGA AAC GAG CGA	
Procollagen 1 (F)	Z78279	AAG GGT GAG ACA GGC GAA CAA	170
Procollagen 1 (R)		TTG CCA GGA GAA CCA GCA GAG	
c-fos (F)	XM_234422	GTG GTG GAA GGC GTA TCG AGT TT	102
c-fos (R)		GTG TGA TGC CAG AAG CAG ATC CA	
c-jun (F)	X17163	CCT AGC TGA ACT GCA TAG CCA GAA	177
c-jun (R)		AAG TTG CTG AGG TTG GCG TAG A	

### cDNA gene expression array analysis

The expression profile of extracellular matrix & adhesion molecules genes was analyzed using the non-radioactive GEArray Q series Mouse gene array (MM-010N, SuperArray Bioscience Corp., Frederick, MD). This array membrane is composed of 96 extracellular matrix & adhesion molecules genes, a plasmid pUC18 negative control, and four housekeeping genes including glyceraldehyde-3-phosphate dehydrogenase (GAPDH), cyclophilin A, ribosomal protein L13a, and β-actin. Biotinylated cDNA probes were denatured and hybridized to extracellular matrix & adhesion molecules gene-specific cDNA fragments spotted on the membranes. After pre-hybridization with GEAhyb Hybridization Solution (SuperArray) of denatured salmon sperm DNA (Invitrogen). The array membrane was hybridized with denatured cDNA probes overnight at 55°C. Following washing the membrane twice with 2 × SSC, 1% SDS and twice with 0.1 × SSC, 0.5% SDS for 15 min at 55°C each, the membrane was blocked with GEAblocking Solution Q (SuperArray) for 40 min and incubated with alkaline phosphatase-conjugated streptavidin for 10 min at room temperature. Chemiluminescent detection was performed using CDP-Star substrate. The results were analyzed with ScanAlyze and GEArray Analyzer. The relative expression levels of different genes were estimated by comparing its signal intensity with that of internal control β-actin.

### Data analysis

RT-PCR results were reported as relative gene expression and the fold change in target genes was determined by 2^-ΔΔCt ^method, where -ΔΔCt = (C_tTarget _- C_tActin_)_GK _- (C_tTarget _- C_tActin_)_control _and Ct value = the cycle number that crosses signal threshold. To evaluate the effect of ABT-627 treatment on gene expression profile in the diabetic group, fold change was determined as (C_tTarget _- C_tActin_)_GK _- (C_tTarget _- C_tActin_)_GK + ABT-627_. Group comparisons (C vs GK or GK vs GK +ABT-627) were performed using Student's t-test. For Array studies, relative gene expression was analyzed using the SAM (Statistical Analysis of Microarrays) software [13].

## Results and discussion

GK rats displayed mildly elevated blood glucose levels that are representative of blood glucose levels seen in patients with Type 2 diabetes (Table 2). There were no significant changes in blood pressure and lipid profile. We have previously shown that GK rats are hypoinsulinemic and display impaired glucose tolerance. Euglycemic hyperinsulinemic clamp studies indicated that GK rats are also insulin resistant. These results strongly suggest that insulin resistance in GKs worsens impaired insulin secretion and leads to hypoinsulinemia as seen in a majority of patients with Type 2 diabetes that eventually become insulin-dependent and thus represent a clinically relevant model.

**Table 2:** Metabolic parameters of Control Wistar and GK rats.

Metabolic Parameters	Control	GK	GK + ABT-627
Body weight (g)	502 ± 11	359 ± 7*	379 ± 29
Blood glucose (mg/dl)	116 ± 5	239 ± 23*	211 ± 18
Mean Arterial Pressure (MAP, mm Hg)	101 ± 2	110 ± 7	112 ± 1

*P < 0.05 vs control

**Figure 1:**
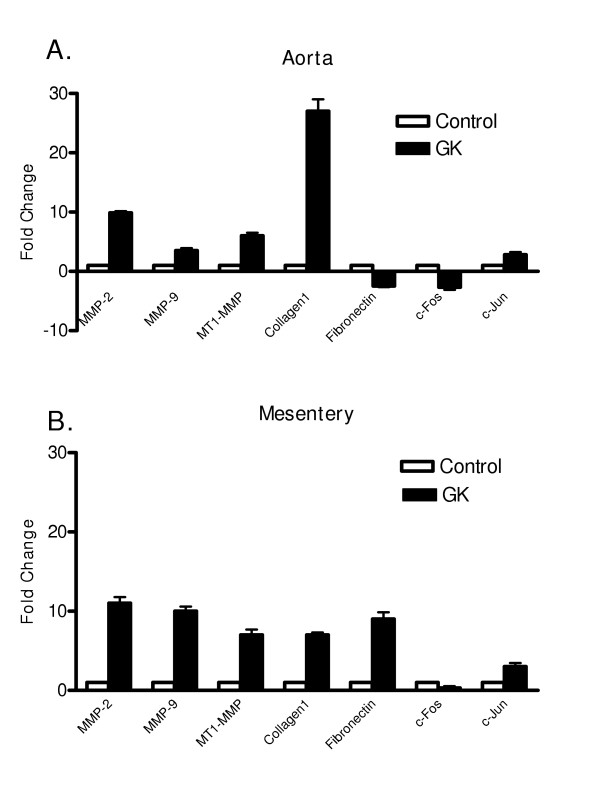
qRT-PCR analysis of diabetes-induced alterations in ECM and MMP expression.

Since MMP-2, MMP-9 and MT1-MMP are major MMP species found in the vasculature and our previous studies suggested time-dependent differential regulation of MMPs in diabetes [8,9,14-16], we first studied MMP and collagen gene expression in aorta and mesentery samples obtained from control and GK rats. Results summarized in Figure 1 demonstrated that mild elevation of blood glucose for 6 weeks is sufficient to stimulate gene expression of MMP-2, MMP-9 and MT1-MMP in both aorta and mesentery samples to a similar degree. Interestingly, collagen type 1 expression was higher in the aortic samples than in mesenteric vessels of diabetic animals. Fibronectin expression was unchanged in aorta but significantly increased in the mesentery. c-jun expression, which is involved in early growth response, was also increased whereas c-fos expression was lower in the diabetic group. To the best of our knowledge, these results are first to demonstrate increased MMP gene expression in small mesenteric arteries and in aorta in diabetes. We have recently reported that MMP protein and activity is increased in the middle cerebral arteries of GK rats and this is associated with vascular remodeling characterized by increased medial thickness and decreased lumen:media ratio [9]. Current findings strongly suggest that changes in enzyme activity parallel changes in gene expression indicating stimulation of the MMP system at transcriptional level in Type 2 diabetes. Our results are consistent with the previous reports that showed increased collagen expression in the mesenteric bed in Type 1 diabetes and further demonstrate that this increase is to a greater extent in large muscular vessels [4,17,18]. Our findings on fibronectin expression in aorta, however, differ from a recent report by Fukuda et al, who demonstrated a 4-fold increase in aortic fibronectin expression [5]. This difference may be attributed to the use of a model of Type 1 diabetes or the time point (26 weeks after the induction of diabetes) at which collagen expression was evaluated.

The role of local vasoactive factors in diabetic vascular remodeling has been an active area of research. Given that hyperglycemia stimulates the production of ET-1, the most potent vasoconstrictor with mitogenic and profibrotic properties, prompted studies with ET receptor antagonists to identify the involvement of the ET system in diabetic vascular complications [4,19-21]. However, a big majority of these studies were conducted using experimental models of Type 1 diabetes or obese animals. We have recently shown that ET-1 levels are elevated in the GK model [9]. Thus, the current study investigated the role of endogenous ET-1 on micro and macrovascular gene expression in the GK model. Results summarized in Table 3 demonstrate that blockade of the ET_A _receptor subtype early in the disease process does not prevent diabetes-induced increases in the expression of ECM proteins and MMPs in aorta. However, ET receptor antagonism completely attenuates increases in MT1-MMP and collagen type 1 expression and partially prevents changes in MMP-2, MMP-9 and fibronectin expression. These novel findings suggest that there is differential regulation of microvascular ECM gene expression in Type 2 diabetes. Our results confirm the findings of Gilbert et al. that ET_A _receptor blockade prevents microvascular hypertrophy in Type 1 diabetes and extends these results to Type 2 diabetes [4].

**Table 3:** qRT-PCR-based fold changes in gene expression in control vs diabetic GK rats treated with or without ABT-627.

Aorta			Mesenteric artery	
	C vs GK	GK vs GK+ABT627	C vs GK	GK vs GK+ABT627
MMP-2	↑10*	No change	↑11*	↓3**
MMP-9	↑3*	No change	↑10*	↓2**
MT1-MMP	↑6*	No change	↑7*	↓7**
COL1A1	↑27*	No change	↑7*	↓8**
Fibronectin	↑1.23	↓1.25	↑9*	↓3**
c-Fos	↓3*	↑2	↓3*	↑2**
c-Jun	↑3*	No change	↑3*	↓5**

* p < 0.05 C vs GK, **p < 0.05 GK vs GK+ABT-627

**Figure 2:**
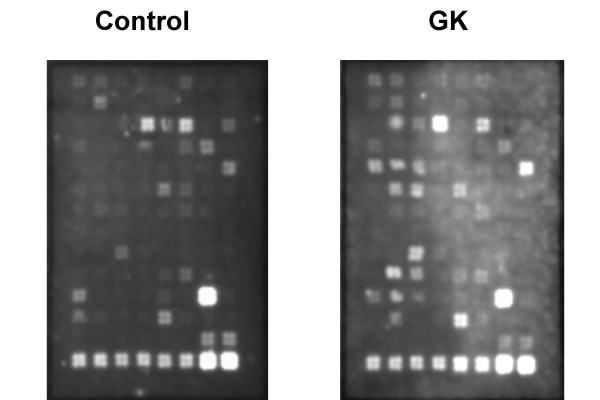
A representative image of extracellular matrix and adhesion molecular microarray of control and GK rats.

In order to determine whether expression of other ECM proteins is altered in the GK model, aortic gene expression profile was investigated using an ECM and Adhesion Molecules pathway specific microarray approach (Fig. 2). Genes that showed a significant change in the expression (p < 0.05 vs control) were grouped as adhesion molecules, ECM proteins and proteases in Table 4 and demonstrated that a total of 27 genes out of 96 genes were differentially regulated in GK rats. There were significant increases in various integrins and MMPs confirming our qRT-PCR results. Increased expression of adhesion molecules is important in diabetes not only to facilitate the adhesion and penetration of inflammatory cells but also to transmit the ECM-generated signals to underlying smooth muscle cells. We observed decreases in thrombospondin-2 (TSP-2) but increases in TSP-3 and -4 expression and no change in TSP-1. These results are particularly interesting with regard to the interaction of TSP-2 with MMP and other ECM proteins [22-24]. TSP-2 belongs to an extracellular glycoprotein family and it is involved in cell-matrix interactions including inhibition of MMP-2 by binding to the active protein as well as decreasing MMP expression [24-26]. TSP-2 knock-out mice display defects in collagen fibril formation and attachment to ECM proteins. In this study we observed decreased TSP-2 and increased MMP-2 activation. Thus, it is reasonable to speculate that decreased TSP-2 expression may contribute to increased MMP-2 activation and decreased cell-matrix attachment resulting in a more free environment for vascular smooth muscle cells to migrate and remodel in diabetes. However, it has to be recognized that current studies involve only gene expression of ECM proteins and should be extended to the protein/activity level to be more definitive.

**Table 4:** Microarray analysis of fold-changes in aortic gene expression in diabetes

Cell Adhesion Molecules		Extracellular Matrix Proteins		Proteases	
Integrin a2	4.1 ↑	Contactin 1	3.5 ↑	MMP2	3.6 ↑
Integrin a2b	4.4 ↑	Col1a1	24 ↑	MMP3	3.1 ↓
Integrin a3	3.3 ↑	Thrombospondin 2	2.2 ↓	MMP9	8.7 ↑
Integrin aL	4.5 ↑	Thrombospondin 3	6.1 ↑	MMP10	7.0 ↑
Integrin aM	5.0 ↑	Thrombospondin 4	4.1 ↑	MMP11	5.2 ↑
Integrin b5	4.4 ↑	Vitronectin	2.3 ↑	MMP19	7.7 ↑
Integrin b6	4.9 ↑			Cathepsin G	2.8 ↑
Ncam	5.8 ↑			Cathepsin E	3.5 ↑
Ncam2	3.0 ↑			uPAR	2.8 ↓
Catenin a-like 1	2.8 ↓				
L-selectin	5.3 ↑				
P-selectin	4.1 ↑				

In conclusion, mild diabetes can stimulate a gene expression pattern that promotes remodeling of both micro and macrovessels. While ET-1 contributes to the alterations in gene expression profile in the microvasculature via activation of the ET_A _receptor subtype, changes in macrovascular gene expression are independent of ET-1.

## Authors' contributions

WeiWei Song performed all the experiments and Adviye Ergul participated in all experimental aspects and preparation of the manuscript.
